# Date of Admission during COVID-19 Pandemic Impacted Patient Outcomes in Addition to the Higher Efficacy of Tocilizumab Plus High-Dose Corticosteroid Therapy Compared to Tocilizumab Alone

**DOI:** 10.3390/jcm11010198

**Published:** 2021-12-30

**Authors:** Moncef Belhassen-García, Antonio Sánchez-Puente, Pedro-Ignacio Dorado-Díaz, Amparo López-Bernús, Jesús Sampedro-Gómez, Raúl Azibeiro-Melchor, Edgard Marcano-Millán, Beatriz Rodríguez-Alonso, María-Elisa Sánchez-Barrado, Ignacio Hernández-García, Ignacio Madruga, Guillermo Hernández-Pérez, Cristina Carbonell, Judit García-Aparicio, Laura Burgos, Eugenia López-Sánchez, Carlos Reina, Ana-María Ramón, Laura Cestero-Ramírez, Fátima Boumhir, Daniel Encinas-Sánchez, María Sánchez-Ledesma, Jacinto Herráez, Patricia Araoz, María-José Sánchez-Crespo, Sandra Rodríguez-Rodríguez, Ana-Elisa Rodríguez-Gude, Miguel-Vicente Sánchez-Hernández, Rafael Borrás, Víctor Sagredo-Meneses, Pedro-Luis Sánchez, Miguel Marcos, José-Ángel Martín-Oterino

**Affiliations:** 1 Infectious Diseases Unit, Department of Internal Medicine, University Hospital of Salamanca-IBSAL, 37007 Salamanca, Spain; mbelhassen@saludcastillayleon.es (M.B.-G.); alopezb@saludcastillayleon.es (A.L.-B.); ghernandezp@saludcastillayleon.es (G.H.-P.); ccarbonell@saludcastillayleon.es (C.C.); 2 Centro de Investigación de Enfermedades Tropicales de la Universidad de Salamanca (CIETUS), University of Salamanca, 37007 Salamanca, Spain; 3 Department of Internal Medicine, University Hospital of Salamanca-IBSAL, University of Salamanca, 37007 Salamanca, Spain; beamedicina@gmail.com (B.R.-A.); imadruga@saludcastillayleon.es (I.M.); jgarciaa@saludcastillayleon.es (J.G.-A.); lmartinez-burgos@saludcastillayleon.es (L.B.); eugeniamlsanchez@gmail.com (E.L.-S.); carlosreinabaez@gmail.com (C.R.); ramon.anamaria@gmail.com (A.-M.R.); fboumhir@saludcastillayleon.es (F.B.); dencinas@saludcastillayleon.es (D.E.-S.); mledesma@saludcastillayleon.es (M.S.-L.); jherraez@saludcastillayleon.es (J.H.); paraoz@saludcastillayleon.es (P.A.); mjsanchezcre@saludcastillayleon.es (M.-J.S.-C.); drusan_13@hotmail.com (S.R.-R.); anaelisarodriguezgude@gmail.com (A.-E.R.-G.); jmoterino@saludcastillayleon.es (J.-Á.M.-O.); 4 Department of Cardiology, University Hospital of Salamanca-IBSAL, University of Salamanca, 37007 Salamanca, Spain; asanchezpu@saludcastillayleon.es (A.S.-P.); pidorado@saludcastillayleon.es (P.-I.D.-D.); jmsampedro@saludcastillayleon.es (J.S.-G.); plsanchez@saludcastillayleon.es (P.-L.S.); 5 Centro de Investigación Biomédica en Red de Enfermedades Cardiovasculares (CIBERCV), Instituto de Salud Carlos III, 28013 Madrid, Spain; 6 Department of Hematology, University Hospital of Salamanca-IBSAL, University of Salamanca, 37007 Salamanca, Spain; raulazi@usal.es; 7 Department of Intensive Care Medicine, University Hospital of Salamanca-IBSAL, University of Salamanca, 37007 Salamanca, Spain; ejmarcano@saludcastillayleon.es (E.M.-M.); lauracr_91@icloud.com (L.C.-R.); vsagredo@saludcastillayleon.es (V.S.-M.); 8 Department of Anesthesiology and Reanimation, University Hospital of Salamanca-IBSAL, University of Salamanca, 37007 Salamanca, Spain; mesanchezba@saludcastillayleon.es (M.-E.S.-B.); mvsanchezher@saludcastillayleon.es (M.-V.S.-H.); 9 Department of Preventive Medicine and Public Health, Lozano Blesa University Clinical Hospital of Zaragoza, 50009 Zaragoza, Spain; ihernandezga@salud.aragon.es; 10 Department of Emergency Medicine, University Hospital of Salamanca-IBSAL, University of Salamanca, 37007 Salamanca, Spain; rborras@saludcastillayleon.es

**Keywords:** COVID-19, SARS-CoV-2, infectious diseases, machine learning, tocilizumab, corticosteroids

## Abstract

Background: The evidence for the efficacy of glucocorticoids combined with tocilizumab (TCZ) in COVID-19 comes from observational studies or subgroup analysis. Our aim was to compare outcomes between hospitalized COVID-19 patients who received high-dose corticosteroid pulse therapy and TCZ and those who received TCZ. Methods: A retrospective single-center study was performed on consecutive hospitalized patients with severe COVID-19 between 1 March and 23 April 2020. Patients treated with either TCZ (400–600 mg, one to two doses) and methylprednisolone pulses (MPD-TCZ group) or TCZ alone were analyzed for the occurrence of a combined endpoint of death and need for invasive mechanical ventilation during admission. The independence of both treatment groups was tested using machine learning classifiers, and relevant variables that were potentially different between the groups were measured through a mean decrease accuracy algorithm. Results: An earlier date of admission was significantly associated with worse outcomes regardless of treatment type. Twenty patients died (27.0%) in the TCZ group, and 33 (44.6%) died or required intubation (*n* = 74), whereas in the MPD-TCZ group, 15 (11.0%) patients died and 29 (21.3%) patients reached the combined endpoint (*n* = 136; *p* = 0.006 and *p* < 0.001, respectively). Machine learning methodology using a random forest classifier confirmed significant differences between the treatment groups. Conclusions: MPD and TCZ improved outcomes (death and invasive mechanical ventilation) among hospitalized COVID-19 patients, but confounding variables such as the date of admission during the COVID-19 pandemic should be considered in observational studies.

## 1. Introduction

Coronavirus disease 2019 (COVID-19) has evolved into a global pandemic with a profound impact on public health. Approximately 20% of cases may develop severe COVID-19 infection with pneumonia, which could potentially lead to hypoxic respiratory failure, acute respiratory distress syndrome, and/or septic shock [1,2]. Patients with severe or critical COVID-19 usually display features of systemic inflammation, with increased levels of proinflammatory cytokines (interleukin (IL)-1 or IL-6) and other acute phase reactants (C-reactive protein (CRP), D-dimer or ferritin [3]). This hyperinflammatory response to SARS-CoV-2 has been suggested to play a key role in the pathogenesis of severe COVID-19, including lung damage and microvascular thrombosis [4].

Accordingly, immunomodulatory drugs such as glucocorticoids have been proposed as a treatment for patients with severe COVID-19 to tackle hyperinflammation and immune dysregulation [5]. Indeed, the use of dexamethasone and other glucocorticoids in severe COVID-19 has been associated with lower mortality in several trials [6]. Although controversial results have been reported, evidence also suggests that the use of interleukin (IL)-6 antagonists, such as tocilizumab (TCZ), is associated with a mortality benefit [7,8]. It is uncertain, however, whether the combined use of TCZ and glucocorticoids improves survival, but limited evidence from observational studies [9] and a subgroup analysis of an open-label trial [10] point toward a potential benefit of this combination. Observational studies, however, have potential limitations, such as differences in patients characteristics between groups, the availability of drugs at any given time, and mortality differences in different time points of the pandemic [11,12]. These limitations may be partially overcome with the use of advanced statistical techniques and previously validated prognostic scores to adjust baseline risk, such as the COVID-19 Salamanca Risk Score [13].

Therefore, the aim of our study was to compare, by means of machine learning methodology, the outcomes of hospitalized patients with COVID-19 who received TCZ compared with those who received TCZ and high-dose glucocorticoid pulse therapy.

## 2. Materials and Methods

### 2.1. Population and Study Design

We performed a retrospective study at a 1000-bed university tertiary care hospital located in Salamanca (northwestern Spain). Consecutive patients admitted due to severe COVID-19 from 1 March to 23 April (peak of the first wave in our center) were analyzed for this study. Patients with COVID-19 were considered to have severe disease because of respiratory failure or other organ dysfunction assessed by Sequential Organ Failure Assessment (SOFA) score > 2, which received per protocol in our center TCZ and/or corticosteroids. TCZ was administered according to our hospital protocol and the treatment consisted of two doses until 26 March (administered at a dosage of 8 mg/kg (max 800 mg) by two consecutive administrations 8 h apart) and one dose thereafter (600 mg (400 mg for weight < 70 kg)), due to the recommendation issued on this date by the Spanish Agency of Medicines and Medical Devices (AEMPS) based on drug shortage. High-dose corticosteroid pulse therapy was administered according to the hospital protocol after 26 March (250 mg of intravenous methylprednisolone (MPD) for 3–5 days) and previously at the physician’s discretion. Patients who received at least 125 mg of intravenous MPD (or dexamethasone equivalent in case the hospital was out of stock) for 2 to 5 days and intravenous TCZ composed the corticosteroid and TCZ (MPD-TCZ) group, and patients who received only intravenous TCZ composed the TCZ group. Patients within these groups were also able to receive hydroxychloroquine and lopinavir/ritonavir according to the protocol. These groups of patients (MPD and MPD-TCZ) were deemed comparable after stratification by our own validated prognosis score [13]. Thus, we proposed the hypothesis that since the choice of treatment was mainly based on the timing of admission and was not dependent on the characteristics of the patient, the effect of the treatment could be compared between both groups. To understand the potential differences between the patients in both treatment groups, we also analyzed baseline characteristics, comorbidities, outpatient treatments, symptoms, admission measures, and laboratory findings at the time of admission for these patients with three approaches: (a) univariate tests between measured variables in both groups; (b) a comparison using a previously validated machine learning prognostic score for the severity of the disease (COVID-19 Salamanca Risk Score [13]); and (c) the development of a newly trained machine learning model aimed at detecting differences between both treatment groups to be used as a multivariate test.

### 2.2. Data Collection

We collected data on demographic variables, the patients’ individual comorbidities and Charlson Comorbidity Index scores, chronic medical treatments, clinical characteristics, physical examination parameters, and the laboratory findings available at hospital admission, as previously described [13]. Regarding patient outcomes, we defined the severity of disease progression during hospitalization as the use of invasive mechanical ventilation or death. Institutional approval was provided by the Ethics Committee of the University Hospital of Salamanca (2020/03/470), and the need for informed consent was waived. All datasets were anonymously analyzed, and the study was performed following the current recommendations of the Declaration of Helsinki [13].

### 2.3. Univariate Analysis

The differences in the measured variables between both treatment groups were analyzed using Student’s *t*-test for continuous variables and Fisher’s exact test for binary variables. If treatment was assigned randomly to all patients and the measured variables were independent, the *p* values of these tests would be distributed uniformly. Consequently, we would expect approximately six variables showing differences with statistical significance (*p* < 0.05) between both treatment groups in univariate analysis. In addition, Kaplan–Meier curves were used to compare mortality rate between groups (long rank test).

### 2.4. Machine Learning Severity Score Comparison by Means of COVID-19 Salamanca Risk Score

The relevance of the collected variables in the prognosis of the patients was previously analyzed in the same cohort of patients [13], where a prognostic score was developed through machine learning to predict severity (defined as death or intubation), regardless of treatment type. The internal validation results evaluated the predictive power with a value of 0.85 of the area under the receiver operating characteristic (ROC) curve [14]. Of note, an external validation using another cohort from the Hospital Clinic of Barcelona, Spain, obtained an area under the receiver operator characteristic (ROC) curve of 0.83, consistent with the internal validation results [13].

As previously described, the final model of this COVID-19 Salamanca Risk Score identified the following variables as predictors of severity in both our cohort and the external validation cohort [13]: the peripheral blood oxygen saturation (SpO_2_)/fraction of inspired oxygen (FiO_2_) ratio, patient age, estimated glomerular filtration rate, procalcitonin levels, C-reactive protein levels, updated Charlson comorbidity index scores, and lymphocyte levels. We considered these predictors separately in the multivariate analysis, as differences in these variables between the treatment groups could have likely impacted our results. Additionally, due to the changing epidemiological situation and evolution of the COVID-19 pandemic, we performed a comparison of the mortality and severity in both treatment groups matched to both the basal risk score of the patients and the date of admission of the patients. This comparison was performed as a subclassification matching with five subclasses divided along quintiles [15], to assess the effects of the confounders within each treatment group, the effect of the treatment within each stratum, the distribution of each group along the subclasses, and the region of common support. A Mann–Kendall test was also performed to detect trends in the outcomes according to the date of admission.

### 2.5. Multivariate Analysis through Machine Learning Methodology

To test the independence between both treatment groups in a multivariate analysis, we developed a machine-learning algorithm for predicting whether a patient belonged to the TCZ or MPD-TCZ group. Machine learning methods were chosen because they encompass classical statistic models (such as logistic regression) while providing the benefits of regularization and cross-validation, and also offer different categories of models of greater discrimination power in case of non-linear relationships and interaction effects between the variables. The performance of this algorithm was given by the area under the ROC curve and the corresponding 95% confidence interval. The null hypothesis (lack of statistically significant differences between the groups) was rejected if the value of 0.5 for the area under the ROC curve fell outside the confidence interval. This analysis was performed both using all clinical variables and using only the variables included in the abovementioned COVID-19 Salamanca Risk Score [13].

In brief, machine learning methodology was described as follows [13]: data from patients in both treatment groups were preprocessed, variables with less than 70% completion were dropped, and missing values were filled using a 10-nearest neighbors algorithm [16]. Three classification algorithms that represented state-of-the-art approaches in machine learning, XGBoost [17], random forest [18], and regularized logistic regression, were trained within a stratified 10-fold cross-validation scheme with 10 repetitions for validation [19]. The evaluation metrics and their confidence intervals were obtained from the testing folds in the cross-validation scheme [20,21]. Additionally, we used a mean decrease accuracy algorithm [18] to identify the most relevant variables in the classification algorithms. In addition to the machine learning classifiers, we tested the discriminatory power of the date of admission, the SOFA score, and the abovementioned machine learning severity score to provide a reference.

## 3. Results

**Figure 1:**
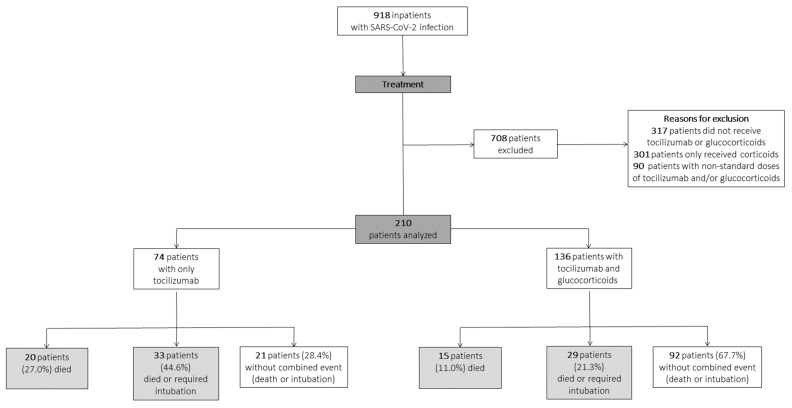
Flowchart of patient selection according to hospital protocol. Patients were included in the tocilizumab and glucocorticoid group if they received at least 125 mg of intravenous methylprednisolone (or dexamethasone equivalent) for 2 to 5 days and one or two doses of intravenous tocilizumab.

**Figure 2:**
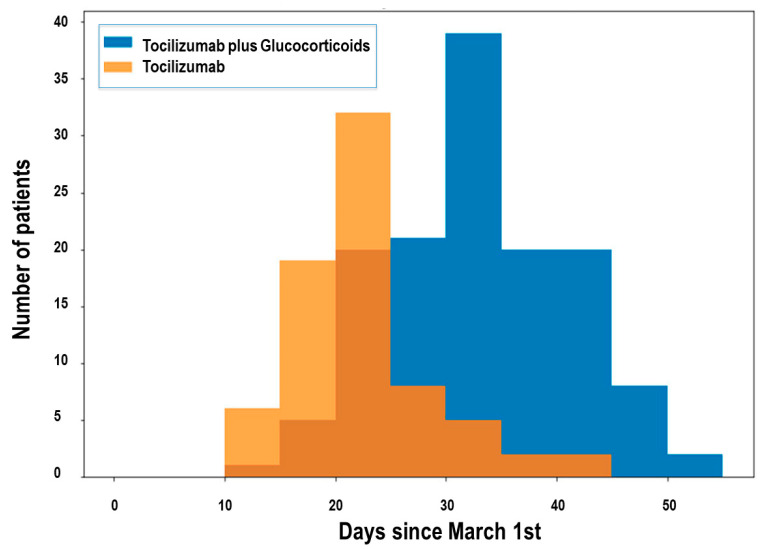
Distribution of patients according to date of admission.

Between 1 March and 23 April 2020, 918 patients were admitted to the University Hospital of Salamanca because of severe COVID-19 with PCR-confirmed infections. Of them, 74 patients received only TCZ, and 136 patients received TCZ and high-dose corticosteroids (126 received MPD and 10 received dexamethasone; Figure 1). The choice of treatment changed with the date of admission, according to modifications in our hospital protocol (Figure 2).

**Figure 3:**
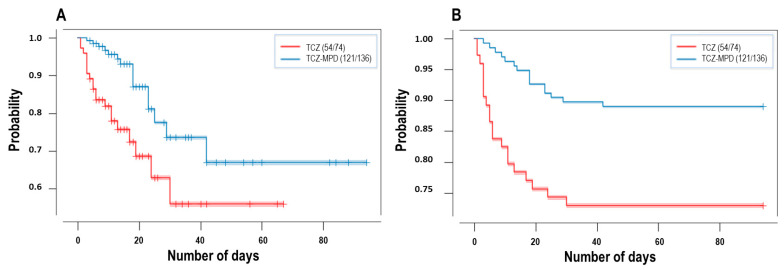
(**A**) Kaplan–Meier survival curves of mortality in admitted patients with severe COVID-19 censoring patients on the date of their discharge (*p* = 0.00195, log-rank test). (**B**) Kaplan–Meier survival curves of mortality in admitted patients with severe COVID-19, without censoring patients (*p* = 0.00151, log-rank test).

A total of 20 patients died (27%) in the TCZ group and the combined event of death and need for invasive mechanical ventilation occurred in 33 patients (44.6%), whereas 15 patients died (11%), and 29 patients died or required intubation (21.3%) in the MPD-TCZ group. These improved outcomes in the MPD-TCZ group were significant after univariate analysis (*p* = 0.006 for mortality and *p* < 0.001 for severity in favor of the MPD-TCZ group). Survival analysis and Kaplan–Meier curves also showed a significant effect (*p* = 0.002) of MPD-TCZ in mortality (Figure 3).

Regarding other differences in patient characteristics between treatment groups, statistically significant differences between the measured variables can be found in Table 1 and Table 2. In summary, differences were found in the following variables: fever, nasal congestion, hemoptysis, the SOFA score, treatment with hydroxychloroquine or azithromycin prior to hospital admission, and serum levels of calcium, magnesium, protein, creatine kinase, and fibrinogen.

The average COVID-19 Salamanca Risk Score, as previously described [13], was 30.7% for the TCZ group and 28.7% for the MPD-TCZ group (*p* = 0.543), which means that patients from both groups have a similar risk of severe outcomes according to this score and therefore that these groups may be considered comparable due to similar baseline risk. In addition, no statistically significant differences were found in any of the variables composing this score between both treatment groups. The comparison of the mortality and severity between the treatment groups matched to the score risk of patients is shown in Table 3. Worse outcomes were associated with higher COVID-19 Salamanca Risk Score in both groups, but patients in the MPD-TCZ group consistently had lower values for death and death or intubation. In addition, and due to the differences in the distribution of patients according to date of admission (Figure 2), data from Table 3 were also matched to date of admission, with the average risk score for each period (as shown in Table 4). After matching these data, we found that both mortality and combined end-point improved over time. Indeed, until 21st March mortality exceeded 30% and combined-end point reached 50% for both TCZ and MPD and TCZ alone but clearly declined thereafter for both groups. The baseline risk as calculated with COVID-19 Salamanca Risk Score of patients treated with tocilizumab also changed over time, although the average prognostic score was similar for both treatment groups. Very few patients were treated with tocilizumab alone after 26th March due to the change in protocol. A Mann–Kendall trend test showed that the combined endpoint events had a decreasing significant trend with admission date in both the TCZ group (*p* = 0.004) and the MPD-TCZ group (*p* = 0.007). Therefore, this finding shows that patients had worse outcomes in the first weeks of the analyzed period regardless of treatment type.

**Figure 4:**
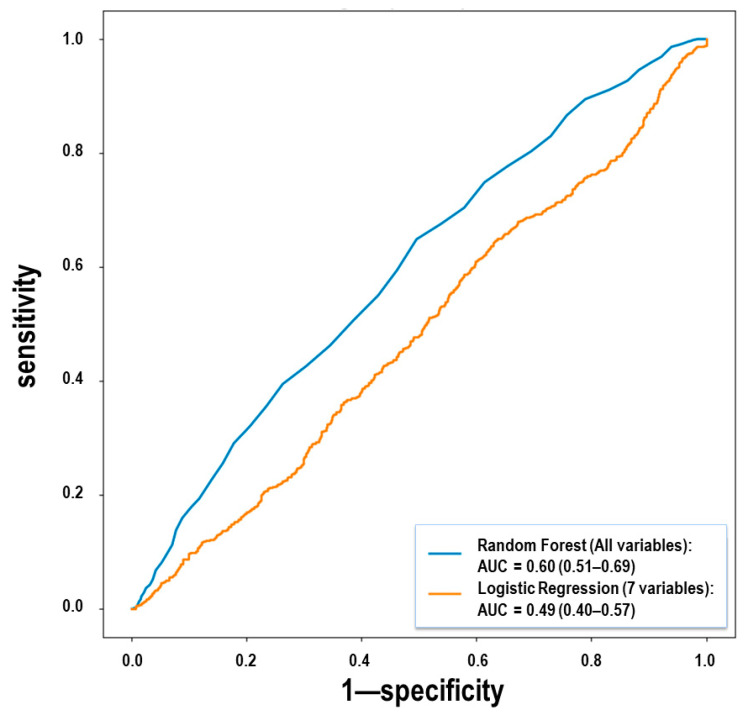
Receiver operating characteristic curves of the different classification algorithms for treatment difference, including best classifier (random forest with all variables) and logistic regression with 7 variables for comparison. The model with all variables was able to show statistically significant differences between both treatment groups but the model constructed with the 7 clinically relevant variables according to the COVID-19 Salamanca Risk Score did not find differences.

Therefore, univariate analysis and stratification according to baseline risk score calculated by means of COVID-19 Salamanca Risk Score and date of admission showed that patients who received tocilizumab and glucocorticoids had better outcomes than those receiving tocilizumab alone, without significant differences in baseline risk score but with differences regarding date of admission. In order to include other variables potentially associated with outcomes, machine learning models were built to test differences between the two treatment groups by means of multivariable analysis. Figure 4 shows the ROC curve for the machine learning models for predicting the treatment group of a patient. The best model built only with the seven variables included in the COVID-19 Salamanca Risk Score (the peripheral blood oxygen saturation (SpO_2_)/fraction of inspired oxygen (FiO_2_) ratio, patient age, the estimated glomerular filtration rate, procalcitonin levels, C-reactive protein levels, updated Charlson comorbidity index scores, and lymphocytes levels) was regularized logistic regression, which obtained an area under the ROC curve of 0.49 (0.40–0.57). However, the best model built with all the clinical variables was the random forest classifier, which obtained an area under the ROC curve of 0.60 (0.51–0.69), thus detecting statistically significant differences between both groups. Therefore, multivariable analysis including all potentially relevant variables confirmed a statistically significant difference between both treatment groups regarding outcomes. The most relevant variables used for this multivariable analysis as calculated by the mean decrease accuracy algorithm can be found in Table 5. Variables that were not included in this table and were not significant in the univariate analysis were less likely to impact mortality in our cohort, regardless of potential differences between groups.

**Figure 5:**
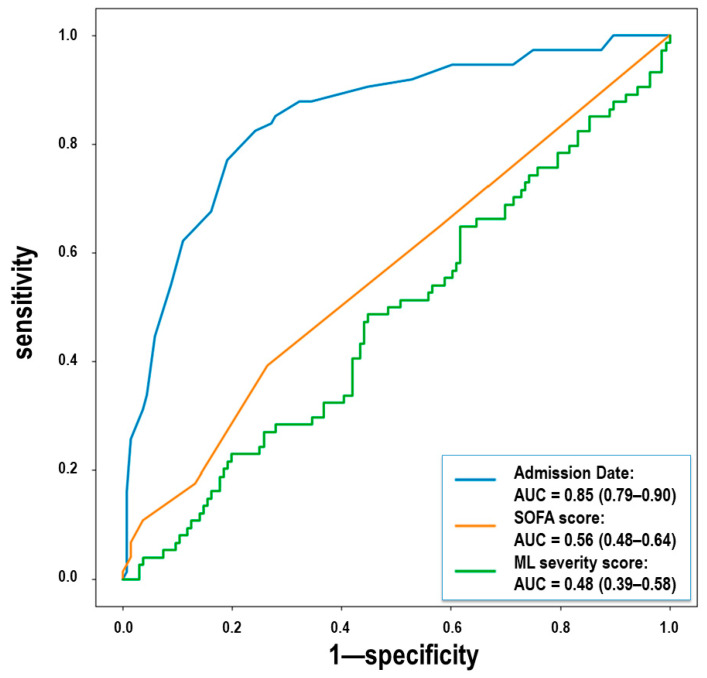
Receiver operating characteristic curves for treatment difference classification based on admission date, SOFA score and machine learning (ML) COVID-19 Salamanca Risk Score. There are no statistically significant differences between both treatment groups according to both prognostic scores, but admission date showed significant discriminant power.

In addition, we compared whether the date of admission was more relevant than prognostic scores to differentiate between treatment groups. Figure 5 shows the ROC curves for the SOFA score, machine learning severity score, and date of admission, when used to distinguish between both treatment groups, showing no discriminant power for the first two and great discriminant power for the latter. Therefore, we confirmed that the date of admission was a significant variable potentially impacting outcome differences between treatment groups.

## 4. Discussion

In our study, we found that the combination treatment with glucocorticoids and TCZ, when compared with TCZ alone, is associated with both reduced mortality and a reduced risk of a composite endpoint of invasive mechanical ventilation and death. To allow comparison, we selected two groups with similar baseline risk according to our previously validated risk score, which made the presence of potential unknown confounders related to patient characteristics unlikely [13]. Additionally, we used a machine learning methodology to assess potential differences between groups after adjusting for other variables, and we found that a random forest classifier including all variables confirmed a significant difference between both treatment groups.

Our results are in line with data from other observational studies showing that this combination therapy is associated with better outcomes when compared with either TCZ or glucocorticoids alone [9,22,23,24]. In addition, subgroup analysis from the Recovery trial [10] found that patients who were receiving glucocorticoids were more likely to benefit from TCZ, and a similar result was reported from a recent meta-analysis including TCZ and other IL-6 antagonists [8]. The efficacy of this combination, however, is not yet fully established due to the lack of randomized trials and the presence of controversial results from other studies (e.g., improved outcomes with TCZ alone instead of with combination therapy [25]). Moreover, previous studies on this topic were prone to potential biases due to small sample sizes and retrospective and observational study designs, despite the different statistical techniques employed to reduce this risk, such as the inverse probability of the treatment weights technique [9] or propensity score matching [24].

In line with this, although we found a significant difference between the treatments after machine learning methodology, we also found that mortality improved over time as an independent variable associated with better outcomes. In our study, the choice of treatment for each patient was made according to the protocol at the time of admission: patients treated in the early stages of the pandemic received only TCZ, while in later stages, the protocol for treatment changed to include both TCZ and corticosteroids. Mortality improvement over time may have therefore been due, at least in part, to different treatment efficacies, as the random forest classifier also confirmed, but a potential confounding effect for the date of admission has to be considered. Indeed, improved mortality over time has already been described in other series of COVID-19 patients and may also be explained by the increased knowledge of the disease, different population groups being affected by the pandemic at different time points, and reduced hospital burden, regardless of treatment type [11]. This would support the hypothesis of the potential role of the date of admission as a confounding variable because such variable would be a risk factor for an effect (e.g., mortality) and would also be associated with the exposure subject of our study in the population from which the effect derives, without being an intermediate step in the causal pathway between the exposure and the effect that we have analyzed [26]. This variable is, however, complex, since a higher mortality risk has also been described at times of high incidence in comparison with inter-wave period [12] and an analysis of epidemiological time delay dynamics showed a marked decrease in the time from hospitalization to death and infection to death during high incidence periods when health care system was under the most pressure [27]. In any case, taking into account this variable could be of great interest since other studies that compared groups of patients with historical controls during the peak of the first pandemic wave have also found worse outcomes for the cohort admitted earlier in the pandemic [22,23]. Considering the striking differences in incidence in different periods of the pandemic, our data, along with the results from other authors, allow us to draw the conclusion that the date of admission should be considered as a variable in observational studies during the COVID-19 pandemic. In addition, this variable should be taken into account in other diseases when relevant temporal trends are present.

Another potential limitation of our study is the different sample sizes between groups and the use of different types and doses of steroids. Although the Recovery trial [6,28] used a low dose (6 mg) of dexamethasone for treatment, other studies, including a small randomized trial, have shown the efficacy of pulse therapy with other steroids [29,30,31], such as MPD (which achieves higher lung tissue concentrations than dexamethasone). Regarding dose, a recent systematic review has shown that COVID-19 patients may be more likely to benefit from medium-high doses of steroids (defined as 90–250 mg/day or 1.5–4 mg/kg/day of MPD or an equivalent dose of other steroids), but higher or lower doses were both associated with improved survival rates [32]. Therefore, despite the lack of evidence regarding an optimal therapy, we do not think that combining the data of patients with equivalent corticosteroid dosages may have a major impact on our results, and other studies have also used this approach [24]. In any case, there is a lack of data on the comparison of corticosteroid pulse therapy with low-dose oral corticosteroids. Finally, although we only present data from the first wave of the pandemic, it is very unlikely to have data to compare tocilizumab vs. tocilizumab and corticosteroids after the first wave because corticosteroids became part of standard treatment in an early phase of the pandemic.

## 5. Conclusions

In conclusion, our data support the potential utility of the combination of glucocorticoids with TCZ, in line with previous findings but outcomes were influenced by date of admission regardless of treatment type and thus this variable as a potential source of bias should be considered in observational studies on this topic. Rigorous randomized trials are needed to assess the efficacy of TCZ and corticosteroids combined and to evaluate the optimal corticosteroid dose for treatment.

## Figures and Tables

**Table 1:** Admission characteristics of patients by treatment group.

	Tocilizumab and Glucocorticoids		Tocilizumab		
**Name**	***n***	**Mean**	***n***	**Mean**	***p*** **-Value**
Age, years (mean ± SD)	136	64.3 ± 11.7	74	65.2 ± 10.9	0.578
Male, *n* (%)	136	92 (67.6%)	74	51 (68.9%)	0.878
**COMORBIDITIES**					
Classic Charlson comorbidity index, *n* (mean ± SD)	136	1.0 ± 1.6	74	0.9 ± 1.3	0.553
Updated Charlson comorbidity index, *n* (mean ± SD)	136	0.6 ± 1.4	74	0.5 ± 1.1	0.616
Myocardial infarction, *n* (%)	136	10 (7.4%)	74	6 (8.1%)	1
Congestive heart failure, *n* (%)	136	7 (5.1%)	74	5 (6.8%)	0.757
Peripheral vascular disease, *n* (%)	136	4 (2.9%)	74	3 (4.1%)	0.699
Arrhythmia, *n* (%)	136	10 (7.4%)	74	3 (4.1%)	0.55
Cerebrovascular accident, *n* (%)	136	5 (3.7%)	74	3 (4.1%)	1
Cognitive impairment, *n* (%)	136	2 (1.5%)	74	3 (4.1%)	0.348
Other central nervous system diseases, *n* (%)	136	3 (2.2%)	74	2 (2.7%)	1
Chronic obstructive pulmonary disease, *n* (%)	136	4 (2.9%)	74	5 (6.8%)	0.284
Asthma, *n* (%)	136	5 (3.7%)	74	2 (2.7%)	1
Other chronic pulmonary disease, *n* (%)	136	7 (5.1%)	74	6 (8.1%)	0.388
Rheumatological disorder, *n* (%)	136	7 (5.1%)	74	3 (4.1%)	1
Peptic ulcer disease, *n* (%)	136	4 (2.9%)	74	2 (2.7%)	1
Hemiplegia/paraplegia, *n* (%)	136	0 (0.0%)	74	1 (1.4%)	0.352
Chronic kidney disease (eGFR < 30), *n* (%)	136	1 (0.7%)	74	1 (1.4%)	1
Solid tumor, *n* (%)	136	11 (8.1%)	74	3 (4.1%)	0.387
Neoplasia without metastasis, *n* (%)	136	10 (7.4%)	74	1 (1.4%)	0.101
Solid tumor metastasis, *n* (%)	136	1 (0.7%)	74	0 (0.0%)	1
Hematologic neoplasm, *n* (%)	136	3 (2.2%)	74	3 (4.1%)	0.427
Chronic acute leukemia, *n* (%)	136	1 (0.7%)	74	2 (2.7%)	0.284
Lymphoma, *n* (%)	136	3 (2.2%)	74	1 (1.4%)	1
Hypertension, *n* (%)	136	55 (40.4%)	74	32 (43.2%)	0.77
Other endocrine disease, *n* (%)	136	12 (8.8%)	74	9 (12.2%)	0.475
Inflammatory autoimmune disease, *n* (%)	136	9 (6.6%)	74	4 (5.4%)	1
Transplant recipient, *n* (%)	136	0 (0.0%)	74	1 (1.4%)	0.352
Obesity, *n* (%)	105	23 (21.9%)	60	19 (31.7%)	0.195
Dyslipidemia, *n* (%)	136	43 (31.6%)	74	34 (45.9%)	0.051
Current smoking, *n* (%)	116	9 (7.8%)	72	4 (5.6%)	0.769
Former/current smoking, *n* (%)	116	33 (28.4%)	72	14 (19.4%)	0.225
Diabetes, *n* (%)	136	31 (22.8%)	74	17 (23.0%)	1
Cancer, *n* (%)	136	13 (9.6%)	74	6 (8.1%)	0.806
**PREVIOUS MEDICATIONS, *n* (%)**					
Angiotensin-converting enzyme inhibitors	135	16 (11.9%)	73	7 (9.6%)	0.817
Angiotensin II receptor blockers	135	23 (17.0%)	71	20 (28.2%)	0.072
Chemotherapy	136	3 (2.2%)	74	0 (0.0%)	0.554
Immunosuppressants	136	4 (2.9%)	73	3 (4.1%)	0.697
Systemic corticosteroids	136	3 (2.2%)	73	4 (5.5%)	0.242
Inhaled corticosteroids	136	6 (4.4%)	73	2 (2.7%)	0.716
Acenocumarol	136	4 (2.9%)	74	1 (1.4%)	0.659
Low-molecular-weight heparin	136	5 (3.7%)	74	3 (4.1%)	1
Direct oral anticoagulants	136	10 (7.4%)	74	5 (6.8%)	1
New oral anticoagulants	136	1 (0.7%)	74	1 (1.4%)	1
Androgen antagonists	136	1 (0.7%)	74	0 (0.0%)	1
Hydroxychloroquine treatment prior to admission, *n* (%)	136	22 (16.2%)	74	5 (6.8%)	0.055
Azithromycin treatment prior to admission, *n* (%)	136	39 (28.7%)	74	10 (13.5%)	0.016
**SYMPTOMS/SIGNS**					
Duration of symptoms before admissionguifen(days), *n* (mean ± SD)	127	7.2 ± 4.5	71	7.2 ± 5.1	0.939
Fever, *n* (%)	136	101 (74.3%)	74	66 (89.2%)	0.012
Duration of fever before admissionguifen(days), *n* (mean ± SD)	90	6.7 ± 4.0	64	6.2 ± 3.5	0.396
Maximum temperature, *n* (mean ± SD)	92	38.3 ± 0.6	60	37.9 ± 4.1	0.357
Dry cough, *n* (%)	136	82 (60.3%)	74	48 (64.9%)	0.554
Productive cough, *n* (%)	136	8 (5.9%)	74	10 (13.5%)	0.072
Chest Pain, *n* (%)	136	15 (11.0%)	74	9 (12.2%)	0.823
Dyspnea, *n* (%)	136	84 (61.8%)	74	53 (71.6%)	0.174
Diminished level of consciousness, *n* (%)	136	9 (6.6%)	74	7 (9.5%)	0.587
Seizures, *n* (%)	136	1 (0.7%)	74	0 (0.0%)	1
Asthenia, *n* (%)	136	55 (40.4%)	74	33 (44.6%)	0.562
Myalgia/arthralgia, *n* (%)	136	28 (20.6%)	74	20 (27.0%)	0.306
Anosmia, *n* (%)	136	6 (4.4%)	74	2 (2.7%)	0.715
Ageusia, *n* (%)	136	9 (6.6%)	74	2 (2.7%)	0.335
Conjunctivitis, *n* (%)	136	1 (0.7%)	74	0 (0.0%)	1
Nasal congestion, *n* (%)	136	1 (0.7%)	74	5 (6.8%)	0.021
Headache, *n* (%)	136	12 (8.8%)	74	6 (8.1%)	1
Odynophagia, *n* (%)	136	5 (3.7%)	74	3 (4.1%)	1
Hemoptysis, *n* (%)	136	0 (0.0%)	74	5 (6.8%)	0.005
Nausea/vomiting, *n* (%)	136	19 (14.0%)	74	6 (8.1%)	0.267
Abdominal pain, *n* (%)	136	7 (5.1%)	74	1 (1.4%)	0.265
Diarrhea, *n* (%)	136	34 (25.0%)	74	15 (20.3%)	0.497
**BASELINE CHARACTERISTICS**					
COVID-19 Salamanca Risk Score (*n*)	136	28.6 ± 23.4	74	30.8 ± 26.0	0.543
SOFA Score (*n*)	136	1.0 ± 1.2	74	1.4 ± 1.6	0.045
Pneumonia, (%)	136	130 (95.6%)	74	70 (94.6%)	0.744
Labored breathing, *n* (mean ± SD)	135	41 (30.4%)	74	26 (35.1%)	0.536
Heart rate, beats/min, *n* (mean ± SD)	136	87.3 ± 16.3	74	89.6 ± 16.2	0.339
Mean arterial pressure, *n* (mean ± SD)	136	87.6 ± 13.6	74	91.5 ± 13.5	0.048
Glasgow Coma Scale, *n* (mean ± SD)	136	14.8 ± 1.0	74	14.8 ± 0.9	0.903
Temperature, *n* (mean ± SD)	136	37.1 ± 0.9	74	37.1 ± 1.1	0.739
SpO_2_/FiO_2_ ratio, *n* (mean ± SD)	136	368.5 ± 90.5	74	342.6 ± 116.6	0.076
Oxygen supplementation, *n* (mean ± SD)	136	67 (49.3%)	74	40 (54.1%)	0.564
Pulmonary infiltrates on chest X-ray, *n* (mean ± SD)	136	131 (96.3%)	74	71 (95.9%)	1
Bilateral pulmonary infiltrate, *n* (mean ± SD)	136	121 (89.0%)	74	65 (87.8%)	0.823
Lopinavir/ritonavir treatment	136	128 (94.1%)	74	72 (97.3%)	0.5

SD: standard deviation; SpO_2_/FiO_2_: arterial oxygen pressure/inspired oxygen fraction; SOFA: sequential organ failure assessment.

**Table 2:** Admission laboratory findings of patients from internal validation cohort by outcome.

Laboratory Findings					
Name	*n*	Mean	*n*	Mean	*p*-Value
Glucose (mg/dL)	128	136.4 ± 61.7	71	126.5 ± 35.2	0.216
Urea (mg/dL)	131	44.1 ± 30.0	72	43.8 ± 27.6	0.936
Urate (mg/dL)	112	4.9 ± 2.0	62	4.8 ± 1.8	0.95
eGFR (mL/min/1.73 m^2^)	133	70.9 ± 21.4	74	71.6 ± 20.8	0.813
Calcium (mg/dL)	125	9.0 ± 0.6	63	8.8 ± 0.5	0.003
Magnesium (mmol/L)	125	2.1 ± 0.3	63	2.0 ± 0.2	0.05
Sodium (mmol/L)	130	136.0 ± 3.3	71	136.5 ± 3.3	0.348
Potassium (mmol/L)	130	4.0 ± 0.5	71	4.0 ± 0.4	0.74
Alanine Aminotransferase (U/L)	129	49.5 ± 73.8	70	41.8 ± 37.7	0.418
Aspartate Aminotransferase (U/L)	107	54.4 ± 46.7	37	68.6 ± 51.3	0.123
Alkaline phosphatase (U/L)	126	87.6 ± 99.0	70	70.3 ± 28.2	0.153
Gamma-glutamyl transferase (U/L)	127	108.8 ± 287.9	70	67.1 ± 52.1	0.232
Lactate dehydrogenase (U/L)	128	394.4 ± 149.9	71	397.0 ± 142.4	0.905
Proteins (g/L)	125	7.6 ± 0.6	64	7.4 ± 0.5	0.012
Albumin (g/L)	122	3.8 ± 0.4	64	3.8 ± 0.4	0.956
Creatine kinase (U/L)	123	140.0 ± 136.3	63	215.1 ± 283.7	0.016
Procalcitonin (ng/mL)	82	0.4 ± 1.1	67	0.6 ± 1.7	0.548
Prothrombine time (%)	116	86.5 ± 16.9	67	84.6 ± 17.6	0.488
INR	115	1.2 ± 0.5	67	1.3 ± 1.2	0.391
Activated partial thromboplastine time (s)	63	34.7 ± 6.8	59	34.6 ± 4.9	0.904
Fibrinogen levels (mg/dL)	112	693.1 ± 192.3	61	626.2 ± 189.8	0.03
Hemoglobin (g/dL)	133	14.3 ± 1.9	72	14.3 ± 1.8	0.954
White blood cells count (×10^9^/L)	116	11.9 ± 35.9	63	7.5 ± 6.3	0.339
Neutrophil cell count (×10^9^/L)	131	6.2 ± 3.2	72	6.2 ± 3.7	0.948
Lymphocyte count (×10^9^/L)	132	3.0 ± 23.3	72	1.2 ± 1.1	0.511
Monocyte count (×10^9^/L)	130	0.5 ± 0.9	71	0.5 ± 0.3	0.667
C-reactive protein (mg/dL)	128	14.6 ± 11.0	71	14.5 ± 11.7	0.947
Interleukin-6 (pg/mL)	25	89.5 ± 104.9	16	183.7 ± 382.0	0.248
D-dimer level (pg/mL)	126	2.2 ± 7.9	64	2.9 ± 11.3	0.617
Platelet count (×10^9^/L)	134	217.8 ± 90.1	74	195.7 ± 79.1	0.079
Bilirubin (total) (mg/dL)	132	0.6 ± 0.3	73	0.6 ± 0.3	0.738
Creatinine (mg/dL)	133	1.1 ± 0.4	74	1.1 ± 0.5	0.973

Variables are presented as the mean ± standard deviation. eGFR: estimated glomerular filtration rate calculated using Chronic Kidney Disease Epidemiology Collaboration (CKD-EPI) equation; INR: international normalized ratio.

**Table 3:** Mortality and severity for each treatment group by risk score classification.

	Tocilizumab Group		Tocilizumab and Glucocorticoids Group		*p*-Value	
Average COVID-19 Salamanca Risk Score	Death or Intubation	Death	Death or Intubation	Death	Death or Intubation	Death
0–8.8%	2/16 = 12.5%	1/16 = 6.3%	3/26 = 11.5%	0/26 = 0%	1.0	0.381
8.8–15.7%	3/11 = 27.3%	2/11 = 18.2%	4/31 = 12.9%	0/31 = 0%	0.353	0.064
15.7–28%	6/16 = 37.5%	2/16 = 12.5%	4/26 = 15.4%	2/26 = 7.7%	0.142	0.628
28–49.5%	9/15 = 60%	4/15 = 26.7%	7/27 = 25.9%	5/27 = 18.5%	0.047	0.698
49.5–100%	13/16 = 81.3%	11/16 = 68.8%	11/26 = 42.3%	8/26 = 30.8%	0.024	0.026
TOTAL	33/74 = 44.6%	20/74 = 27%	29/136 = 21.3%	15/136 = 11%	<0.001	0.006

**Table 4:** Mortality and severity for each treatment group by date of admission.

	Tocilizumab			Tocilizumab and Glucocorticoids		
Date of Admission	Average COVID-19 Salamanca Risk Score	Death or Intubation	Death	Average COVID-19 Salamanca Risk Score	Death or Intubation	Death
March 1st–March 21st	33.2%	22/40 = 55%	13/40 = 32.5%	44.4%	6/12 = 50%	4/12 = 33.3%
March 22nd–March 25th	34.1%	9/21 = 42.9%	7/21 = 33.3%	27.8%	5/21 = 23.8%	2/21 = 9.5%
March 26th–March 31st	25.7%	2/7 = 28.6%	0/7 = 0%	29.9%	7/39 = 17.9%	4/39 = 19.3%
April 1st–April 7th	11.5%	0/4 = 0%	0/4 = 0%	24.4%	5/30 = 16.7%	1/30 = 3.3%
April 8th–April 14th	4.3%	0/2 = 0%	0/2 = 0%	25.9%	6/34 = 17.6%	4/34 = 11.8%
TOTAL	30.8%	33/74 = 44.6%	20/74 = 27%	28.6%	29/136 = 21.3%	15/136 = 11%

**Table 5:** Relative importance of top 10 variables used by the random forest classifier according to mean decrease accuracy algorithm (scaled to the most important one).

Variable	Relative Importance
Mean arterial pressure	1.000
Magnesium levels	0.822
Protein levels	0.759
Lactate dehydrogenase (ldh)	0.651
Sodium levels	0.434
Hemoptysis	0.429
D-dimer levels	0.404
Neutrophil count	0.380
Aspartate aminotransferase (ast) levels	0.370
Lymphocyte count	0.369

## Data Availability

Relevant anonymized patient level data are available on reasonable request. Code to develop machine learning model is already available.
